# Variance Based Measure for Optimization of Parametric Realignment Algorithms

**DOI:** 10.1371/journal.pone.0153773

**Published:** 2016-05-09

**Authors:** Tomislav Milekovic, Carsten Mehring

**Affiliations:** 1 Bernstein Center Freiburg, University of Freiburg, Hansastr. 9A, 79104 Freiburg, Germany; 2 Faculty of Biology, University of Freiburg, 79104 Freiburg, Germany; 3 Department of Bioengineering and Department of Electrical and Electronic Engineering, Imperial College London, South Kensington Campus, SW7 2AZ London, United Kingdom; Monash University, AUSTRALIA

## Abstract

Neuronal responses to sensory stimuli or neuronal responses related to behaviour are often extracted by averaging neuronal activity over large number of experimental trials. Such trial-averaging is carried out to reduce noise and to diminish the influence of other signals unrelated to the corresponding stimulus or behaviour. However, if the recorded neuronal responses are jittered in time with respect to the corresponding stimulus or behaviour, averaging over trials may distort the estimation of the underlying neuronal response. Temporal jitter between single trial neural responses can be partially or completely removed using realignment algorithms. Here, we present a measure, named difference of time-averaged variance (*dTAV*), which can be used to evaluate the performance of a realignment algorithm without knowing the internal triggers of neural responses. Using simulated data, we show that using *dTAV* to optimize the parameter values for an established parametric realignment algorithm improved its efficacy and, therefore, reduced the jitter of neuronal responses. By removing the jitter more effectively and, therefore, enabling more accurate estimation of neuronal responses, *dTAV* can improve analysis and interpretation of the neural responses.

## Introduction

**Fig 1:**
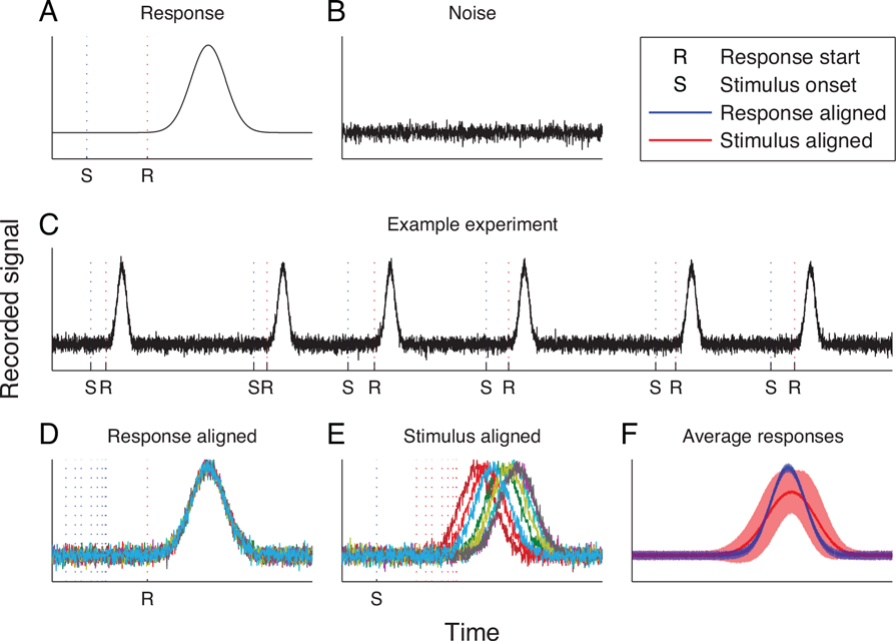
Effect of single trial jitter on the estimation of the underlying neuronal response. A: Neuronal responses are related to the external event (in this example stimulus; S), but are triggered (R) by an internal process, which is not precisely time-locked to the onset of the event. B: A certain amount of noise is recorded together with the relevant neuronal responses. C: During the experiment, the external event occurs multiple times, while the neuronal activity is recorded. D: When neuronal responses are aligned on the response start, the trial average response (F: blue line) is a good approximation of the real neuronal response. However, the response onset is unknown. The trial-averaged response aligned on the event onset triggers (F: red line) does not correctly reproduce the real neuronal response. In addition, the standard deviation across trials calculated using the event onset triggers (F: blue and red shaded tubes) is an incorrect estimate of the variability of neuronal responses. This example was generated using Gaussian white noise with a standard deviation equal to 5% of the maximum response amplitude (SNR = 20). Differences between the response starts and stimulus onsets were modelled using a Gaussian distribution with a standard deviation equal to 1/7 of the response standard deviation (*σ*_*R*_ = 700ms, *σ*_*J*_ = 100ms).

Many neurophysiological studies are investigating neuronal responses to external events. These studies range from simple stimulus evoked neuronal responses in the corresponding primary sensory areas, e.g. neuronal responses to light flashes in the primary visual cortex [1], to neuronal activity correlated to complex behaviours, e.g. neuronal correlates of abstract problem solving [2, 3]. In such studies, neuronal responses are usually extracted by averaging the neuronal signal in order to reduce the “noise”, i.e. parts of the neuronal signal that are not correlated to the stimulus or behaviour that is being investigated. This procedure relies on the assumption that neuronal responses are time locked to the corresponding stimulus or behaviour. This assumption can be challenged, however, as neuronal responses show temporal variability in relation to the corresponding stimulus or behaviour [4–8]. Depending on the amount of the temporal jitter, the underlying neuronal response estimated by averaging may be distorted (Fig 1), possibly leading to mistakes in subsequent analyses and incorrect conclusions.

Several attempts have been made to realign the jittered neural responses using realignment algorithms [9, 10]. By aligning the single trial neural responses, the realignment algorithms can be used to accurately estimate the shape and the variability of the neural response [11], estimate response latencies [12, 13] and enable processing techniques that require single trial responses to be aligned [14].

Typically, parametric realignment algorithms offer a selection of different parameter values, potentially providing greater flexibility in term of neural responses for which the algorithm can be applied to reduce the jitter. However, selecting parameter values suitable for a particular neural response without having accurate knowledge of the response itself can be difficult. Specifically, in situations where the neural recordings are noisy and jittered, accurately estimating the shape of the neural response may not be possible.

Here, we demonstrate that the reduction of variability across trials (difference of time-averaged variance; *dTAV*) can be used as a measure of jitter reduction. This property is crucial for the operation of a parametric realignment algorithm as it can be used to identify a set of parameter values for which the algorithm performed an accurate realignment. We demonstrate this property by comparing the jitter reduction obtained by a parametric algorithm for a range of parameter values versus the jitter reduction obtained using parameter values selected by *dTAV* from the same range. This analysis was performed using an established parametric realignment algorithm [9, 10], referred to as MaxCorr in the rest of the text.

## Methods

The Methods are presented in the following order. First, we describe our simple model of neuronal responses to an external event (stimulus or behaviour). Second, we present the motivation behind the design of the *dTAV* measure using analytic tools. Since the exact triggers of neuronal responses are not known in a real-world application of the algorithm, it is necessary to design such a measure of jitter reduction in order to optimize the parameters of parametric realignment algorithms. Third, we give a brief description of the previously published MaxCorr realignment algorithm [9, 10]. Finally, we describe the details of simulated data used to assess the performance improvements obtained using *dTAV* to optimize the parameters of the MaxCorr realignment algorithm.

### A measure of jitter reduction

We assume that the neuronal signal is a superposition of neuronal responses *r*(*t*) evoked at response onset times *t*_*i*_ plus the Gaussian white noise signal *η*:

$$signal(t)=\sum_{i}r\left(t-t_{i}\right)+\eta \left(t\right)\;\eta \left(t\right)\in N(0,\sigma_{\eta })$$ (1)

where *σ*_*η*_ is the standard deviation of the Gaussian white noise process and *N*(*μ*,*σ*) is a normal distribution with a mean of *μ* and a standard deviation of *σ*. After recording the neuronal signal and if internal neuronal response triggers are known, one can estimate the neuronal response by calculating the response-triggered average $\hat{r}(t)$:

$$\hat{r}(t)=\frac{1}{M}\sum_{i=1}^{M}signal\left(t+t_{i}\right)=r\left(t\right)+\frac{1}{M}\sum_{i=1}^{M}\eta_{i}\left(t\right)\;\eta_{i}\left(t\right)=\eta \left(t+t_{i}\right)$$ (2)

$${\hat{s}}_{\eta }\left(t\right)=\frac{1}{M}\sum_{i=1}^{M}\eta_{i}\left(t\right)\in N\left(0,\frac{\sigma_{\eta }}{\sqrt{M}}\right)$$ (3)

where *M* is the number of responses used to calculate the average; *η*_*i*_(*t*) is the noise in *i*-th trial; and ${\hat{s}}_{\eta }\left(t\right)$ is the random variable drawn from a Gaussian distribution that follows from the presence of noise. In the following derivations, we will use operator *E*( ) for expectation, *V*( ) for variance, $\hat{V}\left(\;\right)$ for sample variance and $\overset{\wedge }{Cov}\left(\;\right)$ for sample covariance. The sample variance of the neural response *r*(*t*) in the presence of the noise is given by:

$$\hat{V}\left(r(t)\right)=\frac{1}{M-1}\sum_{i=1}^{M}{\left(signal\left(t+t_{i}\right)-\hat{r}(t)\right)}^{2}=\frac{1}{M-1}\sum_{i=1}^{M}{\left(\eta_{i}\left(t\right)-{\hat{s}}_{\eta }\left(t\right)\right)}^{2}$$ (4)

and is distributed as a *χ2* distribution [15]:

$$\left(M-1\right)\frac{\hat{V}\left(r(t)\right)}{\sigma_{\eta }{}^{2}}∼\chi_{M-1}^{2}$$ (5)

For a large number of degrees of freedom, i.e. *M* being large, the *χ2* distribution can be approximated by a normal distribution using the following transformation [16]:

$$a∼\chi_{k}^{2}\;\Rightarrow \;\frac{a-k}{\sqrt{2k}}∼N\left(0,1\right)$$ (6)

Using this transformation, Eq 5 becomes:

$$\hat{V}\left(r(t)\right)={\hat{S}}_{V}\left(t\right)\;{\hat{S}}_{V}\left(t\right)\in N\left(\sigma_{\eta }^{2},\sigma_{\eta }^{2}\sqrt{\frac{2}{M-1}}\right)$$ (7)

Experiments are usually designed in such a way that the studied neuronal responses are elicited by events, e.g. sensory stimuli or certain types of behaviour. However, the onset times of the neuronal responses are not known, since these are triggered internally by the brain. The time shift between an event *t*_*Ei*_ and the onset of the neuronal response *t*_*i*_ may not be constant and can be regarded as a stochastic process. In our model, the difference between these two time points is modelled by a Gaussian distribution:

$$t_{i}-t_{E}{}_{i}\in N\left(\mu_{J},\sigma_{J}\right)$$ (8)

where *μ*_*J*_ and *σ*_*J*_ are the mean and the standard deviation of the distribution. If one has access to event times only, as it is the case in a real experiment where the time points *t*_*i*_ when the brain triggers a response are unknown, one can estimate the neuronal response by calculating the event-triggered sample mean ${\hat{r}}_{J}\left(t\right)$:

$${\hat{r}}_{J}(t)=\frac{1}{M}\sum_{i=1}^{M}signal\left(t+t_{E}{}_{i}\right)=\frac{1}{M}\sum_{i=1}^{M}r\left(t+t_{E}{}_{i}-t_{i}\right)+\frac{1}{M}\sum_{i=1}^{M}\eta_{i}\left(t\right)=\bar{r}(t)+{\hat{s}}_{\eta }\left(t\right)$$ (9)

where $\bar{r}(t)$ is the average signal in the presence of the jitter but no noise (*σ*_*η*_ *=* 0). The sample variance of ${\hat{r}}_{J}\left(t\right)$ is given by:

$$\begin{array}{l} \hat{V}\left(r_{J}(t)\right)=\frac{1}{M-1}\sum_{i=1}^{M}{\left(signal\left(t+t_{E}{}_{i}\right)-{\hat{r}}_{J}(t)\right)}^{2}\\ \;=\frac{1}{M-1}\sum_{i=1}^{M}{\left(r\left(t+t_{E}{}_{i}-t_{i}\right)-\bar{r}(t)+\eta_{i}\left(t\right)-{\hat{s}}_{\eta }\left(t\right)\right)}^{2}\\ \;=\frac{1}{M-1}\sum_{i=1}^{M}{\left(\eta_{i}\left(t\right)-{\hat{s}}_{\eta }\left(t\right)\right)}^{2}+\frac{1}{M-1}\sum_{i=1}^{M}{\left(r\left(t+t_{E}{}_{i}-t_{i}\right)-\bar{r}(t)\right)}^{2}\\ \;+2\;\overset{\wedge }{Cov}\left(r\left(t+t_{E}{}_{i}-t_{i}\right),\eta_{i}\left(t\right)\right) \end{array}$$ (10)

The first term of Eq 10 is the sample variance $\hat{V}\left(r(t)\right)$ in the absence of jitter and depends only on the noise *η*_*i*_(*t*) in a way given by Eq 7. The second term is the variance contribution arising from the jitter in the absence of noise and depends only on the jitter. The third term is the sample covariance between the signal and the noise. We can rewrite Eq 10 as:

$$\begin{array}{l} {\hat{s}}_{J}^{2}\left(t\right)=\frac{1}{M-1}\sum_{i=1}^{M}{\left(r\left(t+t_{E}{}_{i}-t_{i}\right)-\bar{r}(t)\right)}^{2}\\ {\hat{S}}_{Cov}\left(t\right)=2\overset{\wedge }{Cov}\left(r\left(t+t_{E}{}_{i}-t_{i}\right),\eta_{i}\left(t\right)\right)\\ \hat{V}\left(r_{J}(t)\right)={\hat{S}}_{V}\left(t\right)+{\hat{s}}_{J}^{2}\left(t\right)+{\hat{S}}_{Cov}\left(t\right) \end{array}$$ (11)

For normally distributed and small jitters *t*_*i*_—*t*_*Ei*_, we can use a Taylor series expansion to express ${\hat{s}}_{J}^{2}\left(t\right)$:

$$\begin{array}{l} \;\bar{r}(t)=\frac{1}{M}\sum_{i=1}^{M}r\left(t+t_{E}{}_{i}-t_{i}\right)\approx \frac{1}{M}\sum_{i=1}^{M}\left(r\left(t\right)+\left(t_{E}{}_{i}-t_{i}\right)\frac{dr\left(t\right)}{dt}\right)=r\left(t\right)+\frac{dr\left(t\right)}{dt}\frac{1}{M}\sum_{i=1}^{M}\left(t_{E}{}_{i}-t_{i}\right)\\ {\hat{s}}_{J}^{2}\left(t\right)=\frac{1}{M-1}\sum_{i=1}^{M}{\left(r\left(t+t_{E}{}_{i}-t_{i}\right)-\bar{r}(t)\right)}^{2}\\ \;\approx \frac{1}{M-1}\sum_{i=1}^{M}{\left(r\left(t\right)+\left(t_{E}{}_{i}-t_{i}\right)\frac{dr\left(t\right)}{dt}-r\left(t\right)-\frac{dr\left(t\right)}{dt}\frac{1}{M}\sum_{j=1}^{M}\left(t_{E}{}_{j}-t_{j}\right)\right)}^{2}\\ \;\approx \frac{dr\left(t\right)}{dt}\frac{1}{M-1}\sum_{i=1}^{M}{\left(\left(t_{E}{}_{i}-t_{i}\right)-\frac{1}{M}\sum_{i=1}^{M}\left(t_{E}{}_{i}-t_{i}\right)\right)}^{2}\\ \;\approx \frac{dr\left(t\right)}{dt}\hat{V}\left(t_{E}{}_{i}-t_{i}\right)\;\Rightarrow \;\frac{\left(M-1\right)}{\frac{dr\left(t\right)}{dt}}\frac{{\hat{s}}_{J}^{2}\left(t\right)}{\sigma_{J}^{2}}∼\chi_{M-1}^{2} \end{array}$$ (12)

For large *M*, we can use transformation (6) and obtain:

$${\hat{s}}_{J}^{2}\left(t\right)\in N\left(\frac{dr\left(t\right)}{dt}\sigma_{J}^{2},\frac{dr\left(t\right)}{dt}\sigma_{J}^{2}\sqrt{\frac{2}{M-1}}\right)$$ (13)

Since *r*(*t + t*_*Ei*_*—t*_*i*_) and the noise *η* are not correlated, the expectation of ${\hat{S}}_{Cov}\left(t\right)$ is zero while its variance depends on the shape of the neuronal response and, thus, cannot be precisely estimated.

Using Eqs 7 and 12, the expectation of $\hat{V}\left(r_{J}(t)\right)$ for large M and small jitters can be expressed as:

$$\text{E}\left(\hat{V}\left(r_{J}(t)\right)\right)=\sigma_{\eta }^{2}+\frac{dr\left(t\right)}{dt}\sigma_{J}^{2}$$ (14)

Our intention was to design a quantitative measure of jitter that could be calculated without knowing the internal triggers of neural responses. Eq 14 provides a direct link between the jitter standard deviation *σ*_*J*_ and the sample variance of the event-triggered response $\hat{V}\left(r_{J}(t)\right)$ which does not require internal neural response triggers. Specifically, Eq 14 shows how comparing $\hat{V}\left(r_{J}(t)\right)$ values may be used to infer which one of any two *σ*_*J*_ values, e.g. *σ*_*J*_*’* and *σ*_*J*_”, is larger than the other. If *σ*_*J*_*’* > *σ*_*J*_”, the corresponding expectations of the $\hat{V}\left(r_{J}(t)\right)$, $\hat{V}{\left(r_{J}(t)\right)}^{\prime }$ and $\hat{V}{\left(r_{J}(t)\right)}^{\prime \prime }$, will follow this relationship:

$$\sigma_{J}^{\prime }>\sigma_{J}^{\prime \prime }\;\Rightarrow \;\text{E}\left(\hat{V}{\left(r_{J}(t)\right)}^{\prime }\right)>\text{E}\left(\hat{V}{\left(r_{J}(t)\right)}^{\prime \prime }\right)$$ (15)

To optimize the alignment, it is necessary to reduce the amount of jitter without the knowledge of the real neuronal response triggers *t*_*i*_. A possible approach minimizes the variance of the stimulus triggered response $\hat{V}\left(r_{J}(t)\right)$ (Eq 10) at one particular time point since a reduction of jitter may result in a decrease of ${\hat{s}}_{J}$ and, hence, lead to a smaller variance (Eq 15). However, since the ${\hat{S}}_{V}$ and ${\hat{S}}_{Cov}$ terms depend on the noise in the signal and, thus, come from a stochastic process, their values might go up by chance and, therefore, mask the reduction of ${\hat{s}}_{J}$. Neighbouring time points of the neuronal response are correlated in time and so is the variance term ${\hat{s}}_{J}$ arising from the jitter. On the other hand, the noise may be correlated on a smaller time scale. Therefore, the stochastic values of ${\hat{S}}_{V}$ and ${\hat{S}}_{Cov}$ may average out across time if the variance is averaged across a sufficiently large time window. A more reliable indicator of jitter reduction may, therefore, be the decrease of the time-averaged variance, *TAV*:

$$TAV=\left\langle \hat{V}\left(r_{J}(t)\right)\right\rangle =\frac{1}{\left(T_{E}-T_{S}\right)}\int_{T_{S}}^{T_{E}}\hat{V}\left(r_{J}(t)\right)dt$$ (16)

As discussed before, averaging over time can reduce the variance of the *TAV*, thereby increasing the reliability of the measure. On the other hand, integrating over periods of time where the neuronal response is small compared to the noise or completely absent may not increase the reliability of *TAV* but instead increase the variance of *TAV*. This trade-off means that the integration window across which the variance is averaged should neither be too long nor too short.

In this study, we used the difference of *TAV*, *dTAV*, as a measure of jitter reduction:

$$dTAV\left(\sigma_{J}^{\prime },\sigma_{J}^{\prime \prime }\right)=TAV\left(\sigma_{J}^{\prime }\right)-TAV\left(\sigma_{J}^{\prime \prime }\right)$$ (17)

We used dTAV to optimize parameters of our realignment algorithm by assuming the following is true:

$$dTAV\left(\sigma_{J}^{\prime },\sigma_{J}^{\prime \prime \prime }\right)>dTAV\left(\sigma_{J}^{\prime },\sigma_{J}^{\prime \prime }\right)\;\Rightarrow \;p\left(\sigma_{J}^{\prime \prime \prime }<\sigma_{J}^{\prime \prime }\right)>p\left(\sigma_{J}^{\prime \prime \prime }>\sigma_{J}^{\prime \prime }\right)$$ (18)

If Eq 18 is correct, we can choose those parameters of our realignment algorithm which lead to the strongest decrease of the time-averaged variability, i.e. we select the parameters for which *dTAV* is largest. While Eqs 15 and 16 indicate that Eq 19 is correct, analytical derivation of such relationship will depend on the neural responses and may not always hold. In the next section, we use numerical simulations to show that such relationship holds for two simulated neural responses that have a mono-phasic and a bi-phasic shape.

### Numerical analysis of the reliability of *dTAV* as a measure of jitter reduction

**Fig 2:**
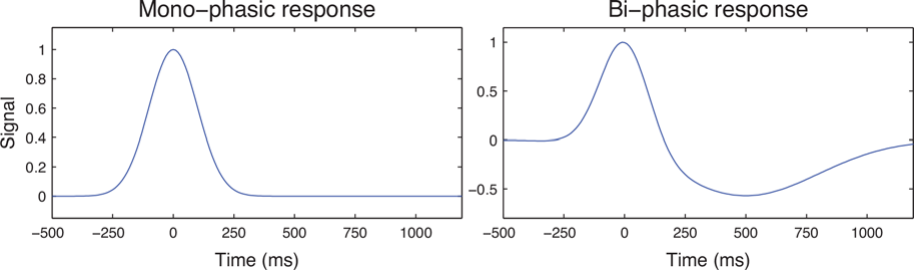
Simulated mono-phasic (left) and bi-phasic (right) neuronal responses.

To demonstrate the conditions for which the *dTAV* is a reliable measure of the reduction in jitter, we performed a set of simulations, each composed of 2000 repetitions of an experiment composed of 100 trials. The neuronal responses were simulated as mono-phasic and bi-phasic functions composed from Gaussian functions (Fig 2):

$$r_{mono}\left(t\right)=\frac{1}{\sqrt{2\pi \sigma_{R}}}e^{-\frac{t^{2}}{2{ο}_{R}^{2}}}$$ (19)

$$r_{bi}(t)=\frac{1}{\sqrt{2\pi \sigma_{R}}}e^{-\frac{t^{2}}{2{ο}_{R}^{2}}}-1.5\cdot \frac{1}{\sqrt{2\pi \cdot 3\sigma_{R}}}e^{-\frac{{\left(t-5\sigma_{R}\right)}^{2}}{2\cdot {\left(3\sigma_{R}\right)}^{2}}}$$ (20)

where *σ*_*R*_ was taken to be 100ms. The shifts of the neural responses, *t*_*i*_*—t*_*Ei*_, were drawn from a Gaussian distribution with zero mean and standard deviation *σ*_*J*_:

$$t_{i}-t_{E}{}_{i}\in N\left(0,\sigma_{J}\right)$$ (21)

Noise was modelled as white Gaussian noise with zero mean and standard deviation *σ*_*η*_ (Eq 1). Our simulations used discrete time with a time step of 1ms. The *TAV* was calculated for each combination of *σ*_*J*_, *σ*_*η*_ and the integration time *T*_*I*_; and for each simulation run *k* using the following equation:

$$TAV_{k}\left(T_{I},\sigma_{\eta },\sigma_{J}\right)=\frac{1}{2T_{I}+1}\sum_{t=-T_{I}}^{T_{I}}{\tilde{V}}_{k}\left(r_{J}(t;\sigma_{\eta },\sigma_{J})\right)$$ (22)

Each simulation was performed by selecting a combination of *σ*_*J*_, *σ*_*η*_ and *T*_*I*_ values. The used *σ*_*J*_ values ranged from 0ms to 120ms in steps of 1ms and the simulation for *σ*_*J*_ = 60ms was performed twice because a dataset with 60ms jitter was used as the starting point for the simulated realignment. *T*_*I*_ values ranged from 30ms to 990ms in steps of 30ms. The *σ*_*η*_ values were selected to model different signal to noise ratios (SNRs), defined as the ratio of the maximum absolute value of the neuronal response and the standard deviation of the noise *σ*_*η*_. We used *σ*_*η*_ values that yielded SNR values of 0.03, 0.05, 0.08, 0.13, 0.20, 0.32, 0.50, 0.79, 1.26 and 2.00 for both mono and bi-phasic responses.

We used these simulations to emulate an experiment where the jitter standard deviation of the dataset was *σ*_*J*_*’* = 60ms before the realignment. This initial dataset was compared to datasets with jitter standard deviations *σ*_*J*_*”* ranging from 0ms (no jitter) to 120ms (doubled jitter) which represented the dataset after the realignment. *dTAV* was then calculated for each combination of *k*, *σ*_*J*_*”*, *σ*_*η*_ and *T*_*I*_.

Ranges of *dTAV* values varied across different orders of magnitude for different *σ*_*η*_ and *T*_*I*_ values. We therefore normalized *dTAV* values by dividing them by the maximum of the absolute value of the 1^st^ and 99^th^ percentile.

$$\begin{array}{l} dTAV_{1\%}\left(T_{I},\sigma_{\eta }\right)=\overset{1\%}{\underset{k,\sigma_{J}^{\prime \prime }}{P}}\left(dTAV_{k}\left(T_{I},\sigma_{\eta },\sigma_{J}^{\prime },\sigma_{J}^{\prime \prime }\right)\right)\\ dTAV_{99\%}\left(T_{I},\sigma_{\eta }\right)=\overset{99\%}{\underset{k,\sigma_{J}^{\prime \prime }}{P}}\left(dTAV_{k}\left(T_{I},\sigma_{\eta },\sigma_{J}^{\prime },\sigma_{J}^{\prime \prime }\right)\right)\\ dTAV_{N}\left(T_{I},\sigma_{\eta }\right)=\max\left(\left|dTAV_{1\%}\left(T_{I},\sigma_{\eta }\right)\right|,\left|dTAV_{99\%}\left(T_{I},\sigma_{\eta }\right)\right|\right)\\ ndTAV\left(T_{I},\sigma_{\eta },\sigma_{J}^{\prime },\sigma_{J}^{\prime \prime }\right)=\frac{dTAV_{k}\left(T_{I},\sigma_{\eta },\sigma_{J}^{\prime },\sigma_{J}^{\prime \prime }\right)}{dTAV_{N}\left(T_{I},\sigma_{\eta }\right)} \end{array}$$ (23)

where $\overset{\text{X}\%}{\underset{a,b}{P}}$ is the *X*-th percentile operator acting over variables *a* and *b*; and max is the maximum value operator. The normalized *dTAV* (*ndTAV*) was binned in 50 equally wide bins spanning the space from -1 to 1. Binned values were used to calculate the probability of jitter reduction, $p\left(\sigma_{J}^{\prime }>\sigma_{J}^{\prime \prime }\right)$, for different *ndTAV* values, while keeping *σ*_*η*_ and *T*_*I*_ constant. To show how the reliability of *dTAV* as a measure of jitter reduction depends on SNR and *T*_*I*_, we calculated the $p\left(\sigma_{J}^{\prime }>\sigma_{J}^{\prime \prime }|ndTAV>0\right)=0.9$ contours in the space spanned by *ndTAV* and *T*_*I*_ for each value of SNR separately. We also calculated the joint probabilities for each combination of *σ*_*η*_ and *T*_*I*_ values, $p\left(\left(\sigma_{J}^{\prime }-\sigma_{J}^{\prime \prime }\right)/\sigma_{J}^{\prime },ndTAV\right)$, in order to verify that the relationship in Eq 18 holds.

### MaxCorr realignment algorithm

We tested whether *dTAV*-based optimization can improve realignment results obtained using previously published MaxCorr algorithm [9, 10]. The MaxCorr algorithm works by approximately maximizing crosscorrelations between each pair of trials in three steps. First, for *N* trials, *N*(*N*-1)/2 crosscorrelations *CX*_*ij*_ for all possible trial pairs *i* and *j* and time lags (*λ*_*i*_- *λ*_*j*_) up to half of the trial length are calculated. Second, a parabolic function *F*_*ij*_(*λ*_*i*_- *λ*_*j*_) is fitted to the crosscorrelation between the *i*-th and the *j*-th trial around the time lag (*λ*_*i*_- *λ*_*j*_)_*MAX*_ for which the crosscorrelation is at maximum.

$$CX_{ij}\left(\lambda_{i}-\lambda_{j}\right)\approx F_{ij}\left(\lambda_{i}-\lambda_{j}\right)=b_{0}+b_{1}\left(\lambda_{i}-\lambda_{j}\right)+b_{2}{\left(\lambda_{i}-\lambda_{j}\right)}^{2}$$ (24)

Parabolic functions *F*_*ij*_(*λ*_*i*_- *λ*_*j*_) are fitted using a neighbourhood (*λ*_*i*_- *λ*_*j*_)_*MAX*_ of several tens of milliseconds. In the last step, all parabolic functions are summed up to derive a new function *F*(*λ*_2_,*…*, *λ*_*N*_), which is quadratic in all of its variables and, therefore, has a unique global maximum (*λ*_2_,*…*, *λ*_*N*_) _*MAX*_. The values of (*λ*_2_,*…*, *λ*_*N*_) _*MAX*_ are calculated by solving the system of linear equations obtained by applying partial derivatives to the parabolic function *F*. (*λ*_2_,*…*, *λ*_*N*_) are used to realign the trials relative to the first trial. MaxCorr algorithm was applied to the same signal used to evaluate the realignment of the *dTAV* algorithm and we evaluated jitter reduction metrics (*σ*_*J*_*’*—*σ*_*J*_*”*) / *σ*_*J*_*’* for both algorithms for comparison.

The implementation of the MaxCorr algorithm in the FIND Matlab toolbox [9] provides the user with the following options and parameters:

1. Maximum allowed correlation time lag (Δ*λ*_*MAX*_). Cross-correletaion is calculated only up to the provided value of the parameter thereby enforcing that all (*λ*_*i*_- *λ*_*j*_)_*MAX*_ values stay within the range of the parameter.
2. Processing of the cross-correlation coefficients. The user can choose whether or not to apply a natural logarithm to the cross-correlation coefficients before fitting the parabolic functions *F*_*ij*_(*λ*_*i*_- *λ*_*j*_).
3. Normalization of cross-correlation coefficients. The user can chose the type of cross-correlation coefficient normalization between: (i) no normalization, which leads to a linear drop of coefficients for larger time lags; (ii) unbiased normalization, which corrects for the linear drop of coeffiecints; and (iii) normalization to autocorrelation, which divides the coefficients with a squared root of a product of autocorrelations of both signals.
4. Iterative jitter reduction. The user can chose to repeat the whole MaxCorr realignment process multiple times (*N*_*REP*_), each time reducing Δ*λ*_*MAX*_ by half.

Different settings of these parameters and options can improve the jitter reduction of a MaxCorr algorithm when applied to a specific dataset. However, a principal way of determining which parameter settings should be used for a given dataset has not yet been investigated. We tested whether *dTAV* can be used to select parameter values that improve the efficacy of the MaxCorr algorithm. I.e., we selected a set of parameters/options, applied the MaxCorr algorithm and calculated *dTAV* and jitter reduction (*σ*_*J*_*’*—*σ*_*J*_*”*) / *σ*_*J*_*’*. After all permutations of the selected parameter values and option settings were used, we selected the (*σ*_*J*_*’*—*σ*_*J*_*”*) / *σ*_*J*_*’* for which the *dTAV* was the highest.

### Simulated data

We used previously published MaxCorr realignment algorithm [9, 10] to investigate whether the *dTAV*–based optimization of parameters can improve its efficacy when performing realignment of simulated single trial neural responses.

We performed 16 types of simulated experiments that differed according to: (i) the number of trials in each experiment between 20, 50, 100 and 200 trials; (ii) the type of the neural response between mono-phasic (*r*_*M*_) and bi-phasic response (*r*_*B*_); and (iii) the distribution of the temporal jitters between a Gaussian distribution with zero mean and 0.1s standard deviation and an uniform distribution spanning -0.2s and 0.2s. For each type of simulated experiments, we performed 100 simulations. In each trial, the single channel neuronal response to an arbitrary stimulus was recorded at 1KHz.

Mono-phasic (*r*_*M*_) and bi-phasic neural responses (*r*_*B*_) were modelled to resemble reported neurophysiological responses as follows (Fig 2):

$$r_{M}(t)=\left\{ \begin{array}{l} e^{\frac{{\left(t-250\right)}^{2}}{2\cdot {\left(83\right)}^{2}}}\;\text{for}\;0\leq t<500\\ 0\;\text{otherwise} \end{array}\right.$$ (25)

$$r_{B}(t)=\left\{ \begin{array}{l} e^{\frac{{\left(t-125\right)}^{2}}{2\cdot {\left(25\right)}^{2}}}\text{-}1.5e^{\frac{{\left(t-250\right)}^{2}}{2\cdot {\left(83\right)}^{2}}}\;\text{for}\;0\leq t<500\\ 0\;\text{otherwise} \end{array}\right.$$ (26)

Neural responses were simulated in the following way:

$$\begin{array}{c} data_{i}(t)=\sum_{j}r\left(t-t_{j}+\left(t_{j}-t_{Ej}\right)\right)+\eta \left(t\right)\\ \eta \left(t\right)\in N(0,\sigma_{\eta ,}{}_{i});\;t_{j+1}-t_{j}\in N(10s,10s)\\ \text{Gaussian distribution of jitters}:\;t_{j}-t_{Ej}\in N(0s,0.1s)\\ \text{Uniform distribution of jitters}:\;t_{j}-t_{Ej}\in U(-0.2s,0.2s) \end{array}$$ (27)

Noise in the recordings was simulated as additive Gaussian noise with zero mean and different standard deviations *σ*_*η*_: 31.62, 19.95, 12.59, 7.94, 5.01, 3.16, 2.00, 1.26, 0.79 and 0.50, which corresponded to SNRs of 0.03, 0.05, 0.08, 0.13, 0.20, 0.32, 0.50, 0.79, 1.26 and 2.00. Temporal distances between the stimulus times were drawn from a Gaussian distribution with a mean of 10s and a standard deviation of 10s. To keep neuronal responses from overlapping, temporal distances below 3s were redrawn. To avoid occasional very large jitters, all jitters with an absolute value above 0.3s were redrawn. The range of the uniform distribution was chosen to roughly contain 95% of the jitters of the Gaussian distribution.

We optimized parameters and options over the following range of parameter values / range of options: (i) Δ*λ*_*MAX*_ = 50ms, 100ms, 200ms, 400ms and 800ms; (ii) processing of the cross-correlation coefficients: “lin” (no processing) and “log” (natural logarithm); (iii) normalization of cross-correlation coefficients: “none”, “unbiased” and “coeff” (normalization to autocorrelation); (iv) *N*_*REP*_ = 1 and 3. *dTAV* was calculated using an integration time *T*_*I*_ of 350ms.

To correctly simulate the outcome of the experiment, we assumed that the person analysing the data would filter the data using a low-pass filter, given that low-frequencies dominate the simulated neuronal responses. We filtered the simulated recordings using 2^nd^ order symmetric Savitzky-Golay filters [17, 18] with different time windows of 100ms, 250ms, 500ms or 1000ms. The time window length of the filter was therefore implemented as an additional parameter optimized in parallel with the MaxCorr algorithm parameters.

## Results

### *dTAV* as a measure of jitter reduction

**Fig 3:**
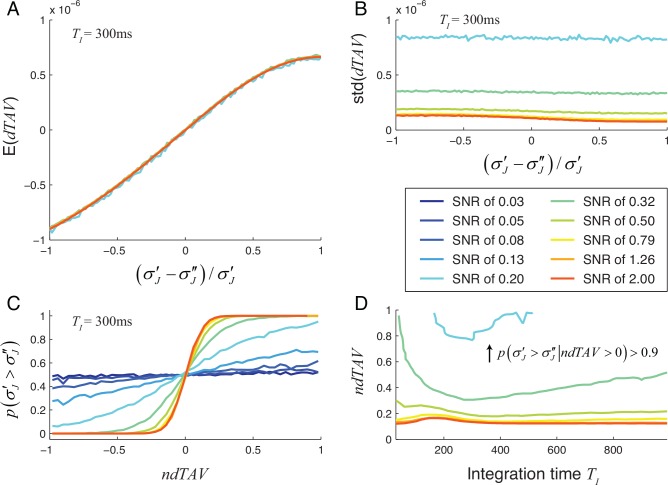
Reliability of *dTAV* as a measure of jitter reduction for mono-phasic neuronal responses. Data obtained by simulating 2000 100-trial experiments. A: Expectation of *dTAV* as a function of the reduction of jitter standard deviation. Lines drawn only for values of SNR of 0.2 and higher. For lower SNR, 2000 repetitions were insufficient to provide a reliable estimate of the expected value of *dTAV* due to the high noise level. For the shown SNR range, the expected value of *dTAV* is independent of the SNR. B: The standard deviation (std) of *dTAV* as a function of the amount of jitter reduction for different SNRs. Standard deviations of *dTAV* for SNR of 0.2 and lower are above 10^−6^ and are, therefore, not shown. C: Probability of jitter reduction as a function of *ndTAV* for different SNRs. Panels A, B and C are shown for integration time *T*_*I*_ of 300ms. D: Values of jitter reduction and integration times for which the probability of correct *dTAV* prediction reaches 90%. For jitter reductions and integration times above the line, the probability for correct *dTAV* prediction, $p\left(\sigma_{J}^{\prime }>\sigma_{J}^{\prime \prime }|ndTAV>0\right)$, is above 90%. For SNRs of 0.13 and lower, the probability of correct *dTAV* prediction never reached 90%.

**Fig 4:**
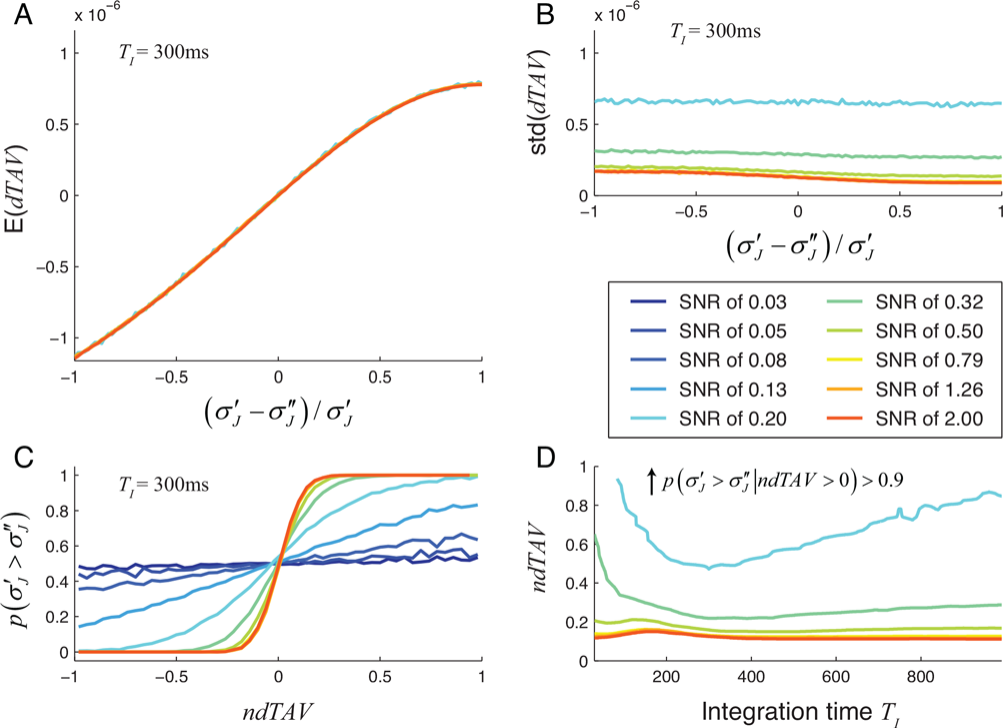
Reliability of *dTAV* as a measure of jitter reduction for bi-phasic neuronal response. See caption of Fig 3 for details.

Using the model of neuronal responses described in section 2.1, we calculated the dependence of *dTAV* on the reduction of the jitter for different SNR levels and integration times *T*_*I*_ by simulating 100-trial experiments 2000 times. Results are summarized in Fig 3 for the mono-phasic signal and in Fig 4 for the bi-phasic signal.

For an integration time of *T*_*I*_ = 300ms, selected for presentation in Figs 3A–3C and 4A–4C, the expectation of the *dTAV* increased monotonously with the amount of jitter reduction and showed little or no dependence on SNR (Figs 3A and 4A). On the other hand, the standard deviation of *dTAV* (Figs 3B and 4B) depended strongly on the SNR and was comparable or larger than the expected value of the *dTAV*. For high SNRs, the expectation of *dTAV* surpassed the standard deviation of *dTAV* even for small reduction in jitter (see Figs 3A, 3B, 4A and 4B). In such cases, *dTAV* is a reliable measure of the amount of jitter reduction, even when the amount of jitter reduction is small.

To measure how well we can rely on the *dTAV* as a measure of jitter reduction, we calculated the probability of correctly predicting that the jitter was reduced based on the normalized *dTAV* values (Figs 3C and 4C). As the SNR was increased, the probability increased up to 1, even for the smallest jitter reductions. For low SNR values the probability never reached 1, even when the jitter was completely removed. To reach a substantial increase of the probability above 0.5, an SNR of about 0.20 or higher was needed.

To provide an insight into the dependence of the probability of correct *dTAV* prediction on the integration time, we calculated the values of integration times and reductions of jitter standard deviation for which the probability of correct *dTAV* prediction reached 90% (Figs 3D and 4D). For high SNRs the performance of the *dTAV* prediction was nearly independent on the integration time whereas for low SNRs the integration time had a stronger influence on the performance of the *dTAV* prediction. The performance of the *dTAV* prediction decreased faster for integration times below the optimal integration time, while it decreased more slowly for integration times bigger than the optimal integration time. Therefore, choosing a short integration time could be more disadvantageous than choosing a longer integration time.

**Fig 5:**
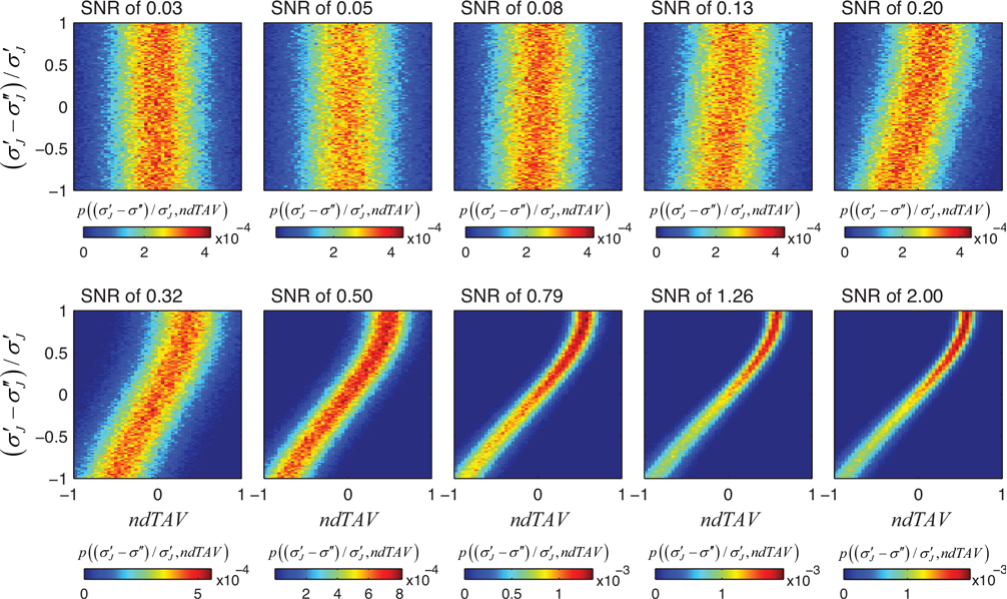
Joint probability distribution of jitter reduction and *ndTAV* for different SNR values for the mono-phasic neuronal response. An integration time window of *T*_*i*_ = 300ms was used. For low SNR, *dTAV* is uninformative as a measure of jitter reduction. As the SNR increases, *dTAV* becomes more informative of the jitter reduction, i.e the ability to differentiate different levels of jitter reduction based on *dTAV* improves substantially.

So far we have computed the probability of jitter reduction given a reduction in *dTAV*. Next, we investigated whether the difference of *dTAV* is also predictor of the expected amount of jitter reduction, i.e. does a larger reduction in *dTAV* also indicate a larger amount of jitter reduction. If so, we can use *dTAV* to optimize parameters of our re-alignment algorithm by selecting parameter values that gave the highest *dTAV* values. To assess whether this is the case, we calculated the joint probability distribution of jitter reduction and *dTAV* for different SNR values for the mono-phasic neural response (Fig 5). For SNRs of 0.13 and lower, *dTAV* provided no or only very little information of the amount of jitter reduction but as the SNR increased (to values of about 0.2 and higher), the relation between *dTAV* and jitter reduction became less variable and *dTAV* became an increasingly good predictor of the amount of jitter reduction. For high SNRs (1.26 and higher), even small differences in *dTAV* indicated increased jitter reduction with high certainty. This suggests that *dTAV* is a good predictor of the amount of jitter reduction for sufficiently high SNRs (of about 0.2 and higher) and can be used to optimize the parameters of our realignment algorithm in such cases. The parameter selection based on *dTAV* will improve with increasing SNR.

### Re-alignment of simulated data using *dTAV* optimized MaxCorr algorithm

**Fig 6:**
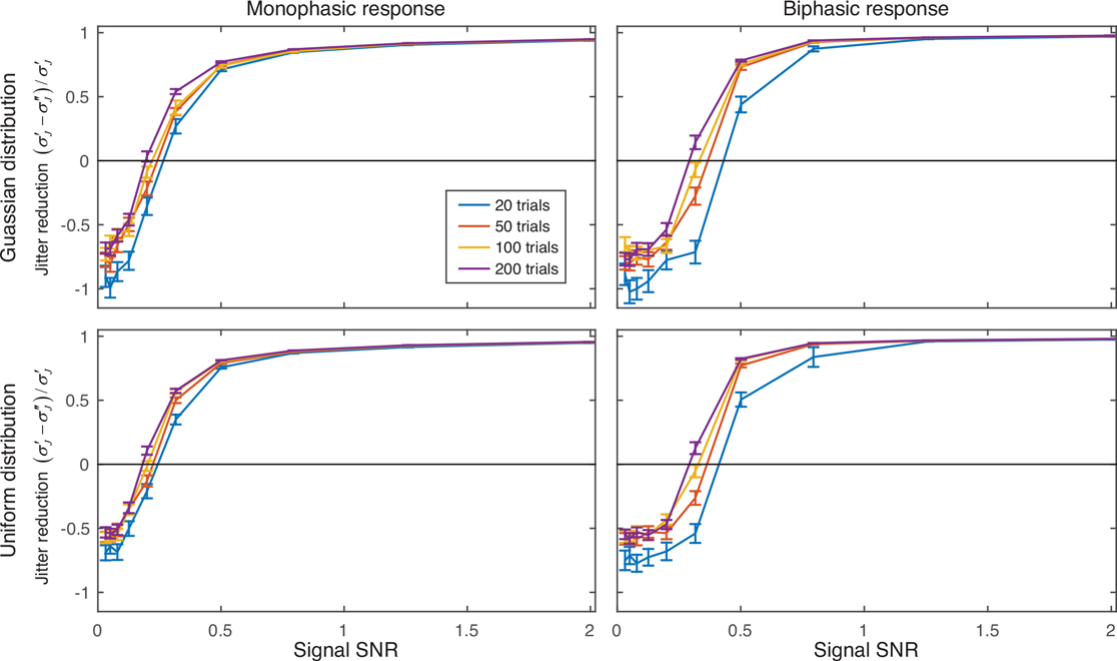
Jitter reduction obtained using *dTAV*-optimized MaxCorr. Jitter reduction is shown for monophasic (left panels) and biphasic (right panels) simulated neural responses and for jitter distributed according to Gaussian (top panels) and uniform distributions (bottom panels). Different lines show jitter reduction for simulated experiments containing different numbers of trials. MaxCorr algorithm options and parameters were optimized over the following values: maximum allowed correlation time lag Δ*λ*_*MAX*_: 50ms, 100ms, 200ms, 400ms and 800ms; processing of the cross-correlation coefficients: “linear” and “logarithmic”; normalization of cross-correlation coefficients: “none”, “coefficient” and “unbiased”; number of consecutive iterations of the MaxCorr algorithm: 1 and 3; and the width of the filter window: 100ms, 250ms, 500ms and 1000ms. *σ’*_*J*_ and *σ”*_*J*_−jitter standard deviation before and after realignment, respectively. All results are averaged over 100 simulation repetitions; error bars depict the standard errors of the mean. For SNR of 1.3 and 2, standard errors are too small to be noticed on the plots.

We used MaxCorr to realign mono-phasic or bi-phasic neuronal responses either for Gaussian or uniform distribution of jitters in 100 simulated experiments containing 20, 50, 100 or 200 trials for different levels of noise (Fig 6). MaxCorr parameters were determined using *dTAV*, which was calculated using an integration window starting at *T*_*S*_ = 0s and ending at *T*_*E*_ = 1s, both in respect to the stimulus times *t*_*E*_. We intentionally did not want to use the results of the *dTAV* reliability analysis (Figs 3D and 4D) since a user of this method would not have the access to such results for his particular neural response. However, we did use the general finding of the *dTAV* reliability analysis that using an integration window wider than the response diminished the *dTAV* reliability less than using a window that is narrower than the response. In other words, we used a window that would certainly be wider than the optimal window, knowing that this does only weakly influence the reliability of *dTAV* as a measure of jitter reduction.

For low SNRs (mono-phasic signal: SNR of 0.20 and lower; bi-phasic signal: SNR of 0.32 and lower) MaxCorr increased the amount of jitter, rather than decreasing it, except in 200-trial simulations with mono-phasic responses and SNR of 0.20, where the jitter was slightly reduced. For intermediate SNRs (mono-phasic signal: SNR of 0.32 and 0.50; bi-phasic signal: SNR of 0.50) MaxCorr reduced the jitter of responses, with its efficacy improving with the number of trials used for realignment. For high SNRs (SNR of 0.79 and higher), MaxCorr removed more than 83% of jitter from the recorded signal, regardless of the number of trials used, distribution of the jitter and the type of response used in the simulations.

To demonstrate that the *dTAV* optimization improves the efficacy of MaxCorr algorithm, we compared the jitter reduction obtained by *dTAV*-optimized MaxCorr with the maximum and the median of jitter reductions obtained using MaxCorr with all parameter sets used for *dTAV*-optimization, as specified in section 2.3. *dTAV* will contribute to the selection of parameter values (i) only if the jitter reduction is higher than the median jitter reduction, which is in some way analogous to random selection of parameter values, and (ii) only if the selected parameters can be used to obtain a large portion of the maximum jitter reduction value.

**Fig 7:**
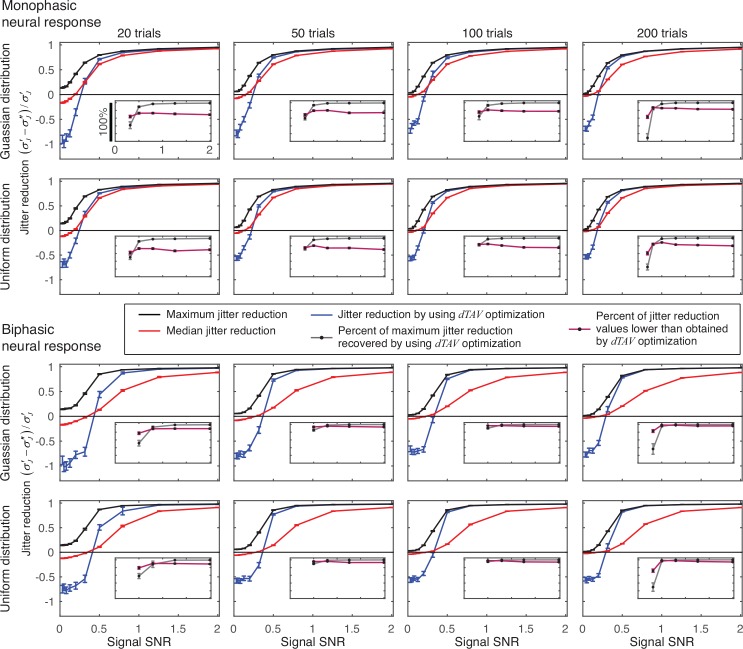
Maximum, median and *dTAV* optimized jitter reduction obtained by MaxCorr algorithm. Main panels show maximum (black lines) and median (red lines) of jitter reduction obtained by using all permutations of options and parameter values used for *dTAV* optimization (see Fig 6 for the list of options and parameter values). Blue lines show reduction of jitter standard deviation obtained using *dTAV* optimized parameter values. Insets show the percentage of maximum jitter reduction recovered when using *dTAV* optimization (grey line with black dots); and the percentage of jitter reduction values, obtained when using all option selections and parameter values, that are lower than the jitter reduction obtained using *dTAV* optimization (purple line with black dots). Both are shown only for SNRs for which the jitter reduction obtained using *dTAV* optimization was positive. All results are averaged over 100 simulation repetitions; error bars depict the standard errors of the mean. For some SNRs standard errors may be too small to be noticed on the plots.

For all SNRs for which *dTAV* optimization of MaxCorr’s options and parameters reduced the jitter, jitter reduction was higher than the median jitter reduction (Fig 7). The proportion of the maximum jitter reduction recovered by the *dTAV* optimized parameters increased with the number of trials in the simulation and with the SNR. For SNR of 0.50 and higher, *dTAV* optimization of MaxCorr’s options and parameters recovered more than 85% of the maximum jitter reduction obtained by MaxCorr using the selected set of parameters, except for a bi-phasic response and 20-trial simulations, where the recovery was still above 50% (Gaussian distribution: 52%; uniform distribution: 58%). For SNR of 0.32 and a monophasic response, the recovery was 42% and 50% for 20-trial simulations for Gaussian and uniform distributions, respectively, and then increased with the increasing number of trials in the experiment up to 84% and 85% for 200-trial simulations for Gaussian and uniform distributions. For a biphasic response and SNR of 0.32 and for lower SNRs for both response types, the recovery is either low or there is even an increase of the jitter standard deviation after the realignment.

To further show that *dTAV* optimization selects efficient sets of parameter values, we counted the percentage of parameter value combinations for which the jitter reduction was lower than that obtained by using *dTAV* optimization, and called it *dTAV* percentile. For all SNRs for which *dTAV* optimization of MaxCorr’s options and parameters reduced the jitter, *dTAV* percentile was higher than 62% (Fig 7 insets). *dTAV* percentiles increase with increasing number of trials (mean *dTAV* percentile: 20 trials: 73%; 50 trials: 79%; 100 trials: 84%; 200 trials: 89%), confirming the property that more trials improve the robustness of the *dTAV* optimization. In addition, *dTAV* percentiles for SNR of 0.50 and higher were higher for biphasic neural response (minimum *dTAV* percentile: monophasic: 67%, biphasic: 77%), implying that *dTAV* optimization improves realignment to a larger extent for a more complex neural responses.

## Discussion

We presented and evaluated a measure for the amount of temporal misalignment between the set of single trial neural responses, called difference of time-averaged variance (*dTAV*), that can be calculated without knowing the internal triggers of neural responses. We derived the relationship between *dTAV* and the precise amount of misalignment, as measured by the standard deviation of the temporal jitter between the trials. Using simulations, we showed that *dTAV* can be used as a measure of misalignment for a wide range of signal-to-noise ratios (SNRs). In addition, we showed that *dTAV* can be used to optimize the parameters of an established MaxCorr realignment algorithm designed to increase the temporal alignment of single-trial neural responses. MaxCorr realignment algorithm was applied to simulated sets of mono and bi-phasic neural responses. We showed that *dTAV* optimization of parameter values of the MaxCorr realignment algorithm leads to substantial efficacy improvements for noise levels up to two and three times larger than the neuronal response amplitudes for mono and biphasic neural response, respectively.

We used mono and bi-phasic potentials composed out of one and two Gaussian functions as examples of neuronal responses. In our simulations, the noise was modelled as Gaussian white noise independent of the simulated neural responses, and the amplitude and shape of the neural responses did not vary across trials. The success of the measure in selecting parameter values that effectively reduce the jitter for intermediate and low noise levels (SNR of 0.32 and higher) and for both mono and bi-phasic neuronal responses, suggests that the *dTAV* measure can be successfully applied in a large number of cases. At the same time, our results show differences between mono and bi-phasic response shapes, suggesting that the signal shape can affect the efficacy of the *dTAV* parameter optimization and, therefore, the performance of the realignment algorithms that use *dTAV*-based parameter optimization.

Our simulations assumed that the recorded neuronal signal is a continuously modulated signal, which is valid for example for local field potentials, electro-corticographic signals (ECoG), electro-/magnetoencephalographic recordings (EEG/MEG) as well as for optical calcium imaging, near-infrared spectroscopy (NIRS) and functional magnetic resonance imaging (fMRI) signals. Furthermore, our measure can also be applied to spike trains by estimating instantaneous neuronal firing from the spike times [19, 20] and using the instantaneous neuronal firing rates as the neuronal signal. In addition, the measure can be applied to continuously modulated signals which were derived from the aforementioned neuronal signals, such as time-resolved spectral amplitudes (e.g. extracted using short-time Fourier transform) or crosscorrelation measures. Misalignment of recordings that depend on other variables than time, such as space or frequency, can also be measured using *dTAV* by exchanging the time variable by the corresponding variable (e.g. spatial coordinate or frequency), thus enabling the effective use of parametric realignment algorithms.

In our study, *dTAV* was defined for one channel of neural signals only. Nonetheless, the measure can easily be extended to multiple channels by summing up *dTAV* of individual channels. MaxCorr algorithm has already been implemented for multiple channels [9, 10]. Therefore, using *dTAV* for MaxCorr parameter optimization in the case of multi-channel neural recordings can be easily achieved.

We show that the performance of MaxCorr algorithm can be improved by testing different parameter sets and calculating *dTAV*. This can be achieved by defining admissible values for each parameter (e.g. within an interval) and scan all possible combinations. If the number of admissible values for each parameter is very high, this process becomes computationally demanding and, therefore, may be time consuming. In our simulations we obtained good realignments within a reasonable timeframe (less than an hour), by optimizing the parameters across a limited number of values. Indeed, our results show (e.g. Figs 3 and 4) that the fine optimization of parameters may not be necessary and the required computational time for parameter optimization may therefore not be problematic.

In general, realignment algorithms can be used to improve the analysis of neurophysiological experiments by improving the estimation of neuronal responses. The obvious case is the estimation of the neuronal response by calculating trial averages. As shown in our example (Fig 1), even if neuronal responses are constant across trials and the noise is uncorrelated to the signal, averaging the jittered single-trial responses can lead to a distorted estimation of the response and the incorrect estimation of the noise. Removing the jitter can improve the estimation of the neuronal response and improves the estimation of the noise. A large number of neuroscience studies investigate neuronal responses related to sensory stimuli. When neuronal responses are well locked to the stimulus [21] realignment methods might be of limited use. On the other hand, neuronal responses may not be locked to the stimulus but, in addition to the stimulus, may also be affected by the internal neuronal state [22, 23] and, therefore be temporally jittered relative to the stimulus onset. Realignment algorithms can be used to find out whether the responses are locked to such internal events and compute the approximate timing of these events. Furthermore, neuronal responses related to behaviour may also be jittered. For example, neuronal responses related to movement planning [24, 25] may be jittered with respect to the times when the movements were initiated if the movements were either self-paced or triggered by another stimulus. Realignment algorithms could be used to align noisy individual trials in order to improve the accuracy of determining the underlying neuronal response (Fig 1). These more accurately determined neuronal responses would then also facilitate a comparison of responses between studies, allowing for identification of neuronal response parts shared between classes of responses. Computing the realignment times may give us also insights into the timing of internal events and into the variability of timings in internal cognitive processes.

Additionally, brain-machine interface systems that detect events based on neuronal recordings [26–33] may benefit from re-alignment algorithms. Such systems require a certain number of trials containing the neuronal responses to calibrate a template that is then used to detect the events from continuous neuronal recordings by matching the current neural activity to the template. If the jitter or the neuronal responses used to calibrate the template is reduced, the template will more closely resemble neural responses to events and, therefore, the detection may perform better.

In summary, we showed that the *dTAV* measure of misalignment can be used to improve the performance of realignment algorithms when applied on simulated neuronal responses for response waveforms commonly observed in neurophysiological recordings and noise levels higher than the neuronal response itself. Hence, the application of the *dTAV* measure and the realignment algorithms that use it to optimize their parameter values can improve analysis and interpretation of neuronal responses and improve the performance of asynchronous detection of events from neuronal recordings.
